# Preserving Leeches

**Published:** 1897-11

**Authors:** 


### Preserving Leeches.

The Pharmacertische Zeitung recommends the following method of keeping leeches: A thin layer of coarse, washed sand is strewed on the bottom of the container, together with some clean, washed straw. This requires renewal every six months; and according to the season, the water (of which about 1 liter suffices for 10 leeches) must more or less frequently be replaced, thoroughly cleaning the jar each time. Treated in this manner, the leeches retain their vitality almost indefinitely, so that a loss is only very rarely met with.—*Practical Druggist.*

